# Green Tea Polyphenols Alleviate Hydrogen Peroxide-Induced Oxidative Stress, Inflammation, and Apoptosis in Bovine Mammary Epithelial Cells by Activating ERK1/2–NFE2L2–HMOX1 Pathways

**DOI:** 10.3389/fvets.2021.804241

**Published:** 2022-01-25

**Authors:** Yanfen Ma, Xuehu Ma, Yanhao An, Yishuo Sun, Wenli Dou, Muyang Li, Hua Bao, Chunhua Zhang

**Affiliations:** ^1^Ningxia Key Laboratory of Ruminant Molecular and Cellular Breeding, School of Agriculture, Ningxia University, Yinchuan, China; ^2^College of Animal Science and Veterinary Medicine, Heilongjiang Bayi Agricultural University, Daqing, China; ^3^Institute of Animal Nutrition and Feed, Inner Mongolia Academy of Agriculture and Animal Husbandry Sciences, Hohhot, China

**Keywords:** oxidative stress, inflammation, apoptosis, green tea polyphenols, bovine mammary epithelial cells, ERK1/2/NFE2L2/HMOX1 pathways

## Abstract

Oxidative stress (OS) is one of the main limiting factors affecting the length of lactation and milk quality in dairy cows. For high-producing dairy cows, the OS of mammary glands is a serious problem. Green tea polyphenols (GTP), found mainly in tea, are a combination of many phenols. GTP have a good effect on antioxidation, inflammation resistance, obesity, fat cell metabolism improvement, and lowering of blood lipid. Therefore, we studied the role of GTP on OS in dairy cows and further investigated whether GTP alleviates oxidative damage of bovine mammary epithelial cells (BMECs) induced by hydrogen peroxide (H_2_O_2_) and its underlying molecular mechanism. In this study, 500 μM of H_2_O_2_ for 12 h incubation was chosen as the condition of the OS model of BMECs. In addition, the present results found that treatment with GTP alleviated the oxidative damage induced by H_2_O_2_ [the activities of superoxide dismutase (SOD), glutathione peroxidase (GSH-Px), and catalase (CAT) were significantly increased, and the contents of malondialdehyde (MDA), 8-isoprostaglandin (8-iso-PG), 8-oxo-deoxyguanosine (8-OHdG), and protein carbonyl (PC) and caspase-3 and caspase-9 activities were significantly reduced]. These effects are related to the activation of the erythrocyte-derived nuclear factor 2-like protein 2 (NFE2L2) signaling pathway and the inactivation of the caspase/Bcl-2 apoptotic pathway. When NFE2L2 short interfering RNA (siRNA) was used to downregulate the expression of NFE2L2 in cultured BMECs, NFE2L2-siRNA transfection abolished the protective effect of GTP on H_2_O_2_-induced intracellular reactive oxygen species (ROS) accumulation and apoptosis. In addition, the mitogen-activated protein kinase (MAPK) inhibition test further proved that GTP relieved H_2_O_2_-induced oxidative damage by activating the NFE2L2 signaling pathway, which was achieved by activating the extracellular-regulated kinase 1/2 (ERK1/2) signaling pathway. Overall, the results indicate that GTP has a beneficial effect on the redox balance of BMECs. In addition, GTP might be a latent antioxidant *in vivo*, which can be administered to ruminants during stressful periods such as the perinatal period.

## Introduction

Dairy cows are one of the most economically important animals, and they produce almost all the dairy products in the daily diet of human beings. Therefore, it is extremely important to ensure the healthy growth and lactation of dairy cows. However, lactating dairy cows often suffer from oxidative stress (OS). Continuous OS not only damages the health of bovine mammary cells but also affects milk production and the quality of dairy products (1). Therefore, reducing and preventing OS of bovine mammary cells is of great significance for promoting the healthy lactation of dairy cows.

For lactating cows, mammary epithelial cells undergo high-intensity metabolism, and produce a large amount of reactive oxygen species (ROS), which can easily cause inflammation. OS occurs when the ROS produced in the system exceed the system's capacity to neutralize and eliminate them (2). OS can affect the lactation time and milk quality of dairy cows (3). Compared with those of healthy-yield dairy cows, the mammary tissues of high-producing cows pass more blood flow, which makes their mammary epithelial cells more prone to OS. OS can induce apoptosis of mammary epithelial cells in dairy cows. Studies have shown that excessive ROS activates the caspase signal promoting transcription-related genes of apoptosis and leading to apoptosis (4). In addition, Miranda et al. (5) found that the cellular antioxidant status can regulate the apoptosis of bovine mammary epithelial cells (BMECs) under OS (5). Therefore, the inhibition of OS is essential in preventing apoptosis in BMECs (5, 6).

The erythrocyte-derived nuclear factor 2-like protein 2 (NFE2L2) signaling pathway plays an important role in protecting cells from oxidation and external damage (7). NFE2L2 is the main regulator of cellular redox balance in non-ruminant animals. It can induce the expression of antioxidants, detoxification enzymes, and downstream phase II enzymes such as superoxide dismutase (SOD) and glutathione peroxidase (GSH-Px) (8, 9). Previous studies suggested that activating the NFE2L2-ARE signaling pathway can protect BMECs from hydrogen peroxide (H_2_O_2_)-induced OS, which indicates that the NFE2L2 pathway may be a potential therapeutic target for protecting the udder tissue of dairy cows from OS (10, 11).

Green tea polyphenols (GTP) are a general term for polyphenol compounds in green tea. Studies have suggested that GTP has good effects on antioxidation, anti-inflammation, cancer prevention, and lipid metabolism regulation (12). Studies have also shown that GTP is a very effective NFE2L2 activator, which can reduce BMECs OS induced by H_2_O_2_ (10, 11). However, whether GTP can alleviate H_2_O_2_-induced BMECs apoptosis and inflammation and its molecular mechanism are still unclear. Therefore, the purpose of this study is to study the therapeutic potential of GTP on BMECs during OS and the underlying molecular mechanism.

## Materials and Methods

### Materials

GTP, ≥98% [high-performance liquid chromatography (HPLC)], was purchased from Sigma-Aldrich (Oakville, Ontario, Canada). PD98059, an inhibitor of extracellular-regulated kinase 1/2 (ERK1/2), was obtained from Sigma-Aldrich (Oakville, Ontario, Canada).

### Cell Culture and Treatment

The BMECs were obtained from the School of Agriculture, Ningxia University. Cells were maintained in Dulbecco's modified Eagle's medium (DMEM) containing 10% fetal bovine serum, 2 mmol/L glutamine, and 1% penicillin/streptomycin at 37°C under a 5% carbon dioxide (CO_2_) cell culture incubator in a 25 cm^2^ cell culture flask. After the cells reached 80–90% confluence, they were removed with 0.25% trypsin/EDTA and transferred to a new 25 cm^2^ cell culture flask.

In order to determine the concentration and time of H_2_O_2_, the BMECs were seeded at a density of 2 × 10^6^ cells per well for 96-well-plate. After incubation for 12 h, the original culture medium was discarded. The control group was added with DMEM complete culture medium, and the other groups were added with DMEM complete medium containing different concentrations of H_2_O_2_ (0, 250, 500, and 1,000 μM); each group had five parallel wells, each well was 100 μl, and the culture was continued for 0, 6, 12, and 24 h. The BMECs viability was measured using a Cell Counting Kit-8 (CCK-8) assay kit (Dojindo, Kumamoto, Japan) according to the manufacturer's instructions. Briefly, 2 × 10^6^ BMECs in 100 μl culture media were plated to a 96-well-plate in suspension. Then, the samples were incubated with a CCK-8 solution (10 μl) for 4 h at 37°C. The absorbance at 490 nm was determined with a microplate reader (Molecular Devices, Sunnyvale, CA). Cell viability was calculated according to the manufacturer's instructions.

In order to determine the safety concentration and time of GTP, the BMECs were seeded at a density of 2 × 10^6^ cells per well for 96-well-plate. After incubation for 12 h, the original culture medium was discarded, adding DMEM complete media containing different concentrations of GTP (0, 20, 50, 100, 200, and 500 μg/ml), and the culture was continued for 0, 6, 12, and 24 h. The BMECs viability was measured using a CCK-8 assay kit (Dojindo, Kumamoto, Japan) according to the manufacturer's instructions. Briefly, 2 × 10^6^ BMECs in 100 μl culture media were plated to a 96-well-plate in suspension. Then, the samples were incubated with a CCK-8 solution (10 μl) for 4 h at 37°C. The absorbance at 490 nm was determined with a microplate reader (Molecular Devices, Sunnyvale, CA).

To establish OS, different treatments were carried out on BMECs. The control group was added with DMEM complete culture medium and cultured for 24 h. The H_2_O_2_ treatment group was added with DMEM complete culture medium to culture for 12 h and then added with 500 μM H_2_O_2_ to culture for 12 h. The GTP treatment group was added with DMEM complete culture medium to culture for 12 h and then added with 100 μg/ml GTP to culture for 12 h. The GTP + H_2_O_2_ treatment group was added with DMEM complete medium containing 100 μg/ml GTP to culture for 12 h and then added with 500 μM H_2_O_2_ to culture for 12 h. BMECs were preincubated for 30 min with or without PD98059 (20 μM) and then cultured with or without GTP (100 μg/ml) for another 12 h followed by H_2_O_2_ (500 μM) exposure. There were five replicate cultures for each treatment in each experiment.

### NFE2L2 Small RNA Interference

Both the short interfering RNA (siRNA) targeting the NFE2L2 coding region and the scrambled non-targeting negative control were designed and synthesized by Shanghai Shenggong Biological Company. The sense of NFE2L2-siRNA primer sequence is TTGCTCAAAGAAAGAGGAGAA, and the antisense is TTCTCCTCTCTTTCTTTGAGCAA. The manufacturer's instructions to transfect BMECs in an antibiotic-free medium was followed. Briefly, BMECs were digested with trypsin and resuspended to 2 × 10^6^ cells/ml in a basal medium without penicillin/streptomycin and cultured at 37°C for 24 h. Lipofectamine 3000 and siRNA were mixed, incubated at room temperature for 20 min, and then transferred to the cell culture plate. After 12 h of transfection, the basic medium was changed with the same fresh medium. After 36 h, the siRNA was removed from the each well, and the cells were collected for experimental analysis.

### Cell Viability Assay and Determination of OS Indicators

According to the instructions, the BMECs viability was evaluated using the CCK-8 kit (Dojindo, Kumamoto, Japan). The activities of SOD, GSH-Px, and catalase (CAT) and the contents of 8-isoprostaglandin (8-iso-PG), protein carbonyl (PC), 8-oxo-deoxyguanosine (8-OHdG), and malondialdehyde (MDA) in BMECs were determined using the enzyme-linked immunosorbent assay (ELISA) kits of Nanjing Jiancheng Institute of Biotechnology (Nanjing, China) according to the manufacturer's instructions. The detection limits of 8-OHdG and 8-iso-PG are 10–300 and 5–150 ng/L, respectively. The contents of caspase-3 and caspase-9 in BMECs were determined using ELISA kits (Nanjing Jiancheng Institute of Biotechnology) according to the manufacturer's instructions.

### Intracellular ROS Detection and Measurement of Cell Apoptosis

Intracellular ROS was measured by dichlorofluorescein staining assay. Briefly, BMECs were washed with phosphate-buffered saline (PBS) and incubated with fresh DMEM containing 10 μM dichlorofluorescein at 37°C for 35 min; then 1 × 10^6^ cells were harvested and suspended in PBS. The optical density at 450 nm was recorded with a microplate reader (Molecular Devices). In addition, cell apoptosis was detected using an annexin V–fluorescein isothiocyanate (FITC)/propidium iodide (PI) apoptosis detection kit (BD Pharmingen, San Jose, CA, USA) according to Wu et al. (13). Briefly, the cells were resuspended in 1× binding buffer and stained with annexin V–FITC as recommended by the manufacturer. The cells were also stained with PI to detect necrosis. Then the cell suspension was ready for analysis by the flow cytometry (Becton Dickinson, Accuri C6 Plus, CA, USA).

### RNA Isolation and Quantitative Real-Time PCR (qRT-PCR)

According to the manufacturer's instructions, TRIzol (Tiangen Biotech Co., Ltd., Beijing, China) was used to isolate total RNA from the cells. First-strand cDNA synthesis and qRT-PCR were performed using the previously described protocol of Ma et al. (10). Briefly, a THUNDERBIRD SYBR qPCR mix kit (TOYOBO) was used for qRT-PCR, and the PCR mixture (final volume 20 μl) includes 10 μl a THUNDERBIRD SYBR qPCR mix, 1 μl forward and reverse primers at 10 mM, 1 μl cDNA, and 7 μl diethyl pyrocarbonate (DEPC)-treated water. The reaction conditions were as follows: 95°C for 3 min, followed by 40 cycles at 95°C for 15 s and at 60°C for 1 min. All qRT-PCRs were performed at least three times. With the Primer 5.0 program used to design gene-specific primers, the relevant sequences are shown in Table 1. The qRT-PCR data were converted to Ct values. The gene expression level was calculated using the 2^−ΔΔCt^ method and normalized using β-actin expression.

**Table 1:** Primer sequences of the genes.

**Gene name**	**Forward primer (5′ → 3′)**	**Reverse primer (5′ → 3′)**	**Accession number**
NFE2L2	CCAGCACAACACATACCA	TAGCCGAAGAAACCTCATT	NM_001011678.2
HMOX1	CAAGGAGAACCCCGTCTACA	CCAGACAGGTCTCCCAGGTA	NM_001014912.1
NQO1	AACCAACAGACCAGCCAATC	CACAGTGACCTCCCATCCTT	NM_001034535.1
Bax	TGGACATTGGACTTCCTTCG	CCAGCCACAAAGATGGTCAC	NM_173894.1
Bcl-2	GGATGACCGAGTACCTGAACC	GCCCAGATAGGCACCCAG	NM_001166486.1
Caspase-3	ACCAACGGACCCGTCAATTT	TCAGCACCACTGTCTGTCTCA	NM_001077840.1
Caspase-9	CTCTGGACTGCTGCATGGTG	TCTCTCGACGGACACAGGAC	NM_001205504.2
β-Actin	GAGAAGATCTGGCACCACTCCT	TCTTCTCCCTGTTGGCCTTGG	DQ066897.1

### Statistical Analysis

Data were analyzed by analysis of variance (ANOVA) and Turkey's test using software SPSS 20.0 (SPSS, Chicago, IL, USA). The data were expressed as mean ± standard deviation (SD), and *P* < 0.05 was considered to be statistically significant. All experiments were replicated five times.

## Results

### Screening for H_2_O_2_ Optimum Conditions

**Figure 1:**
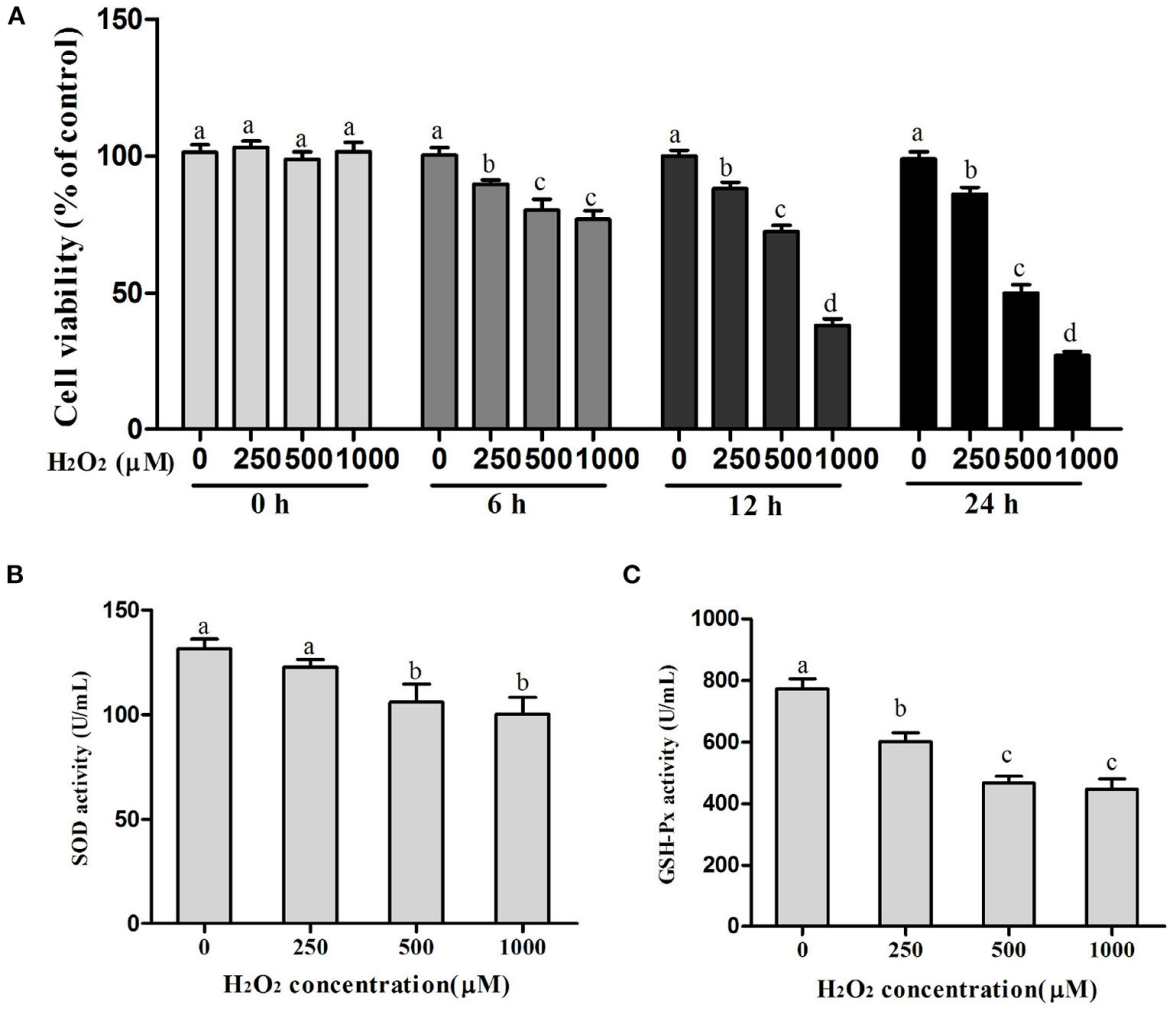
Selection of the optimum conditions for H_2_O_2_ treatment. **(A)** Cell viability in BMECs, **(B)** SOD activity, and **(C)** GSH-Px activity. a,b,c,d, the adjacent lowercase letters indicate significant difference, the alternate lowercase letters indicate extremely significant difference, and the same lowercase letters indicate insignificant difference.

As shown in Figure 1, treatment of BMECs with increasing concentrations of H_2_O_2_ (0–1,000 μM) for 0 to 24 h inhibited time- and dose-dependent cell viability. In addition, with the increase in H_2_O_2_ concentration, SOD and GSH-Px activities decreased significantly. Compared with the control group, the treatment of cells with 500 μM of H_2_O_2_ for 12 h decreased cell viability to 30% (*P* < 0.05) and SOD and GSH-Px activities to 19% (*P* < 0.05) and 40% (*P* < 0.01), respectively. Thus, 500 μM of H_2_O_2_ for 12 h incubation was chosen as the condition of the OS model of BMECs.

### Screening for Optimum Conditions of GTP Incubations

**Figure 2:**
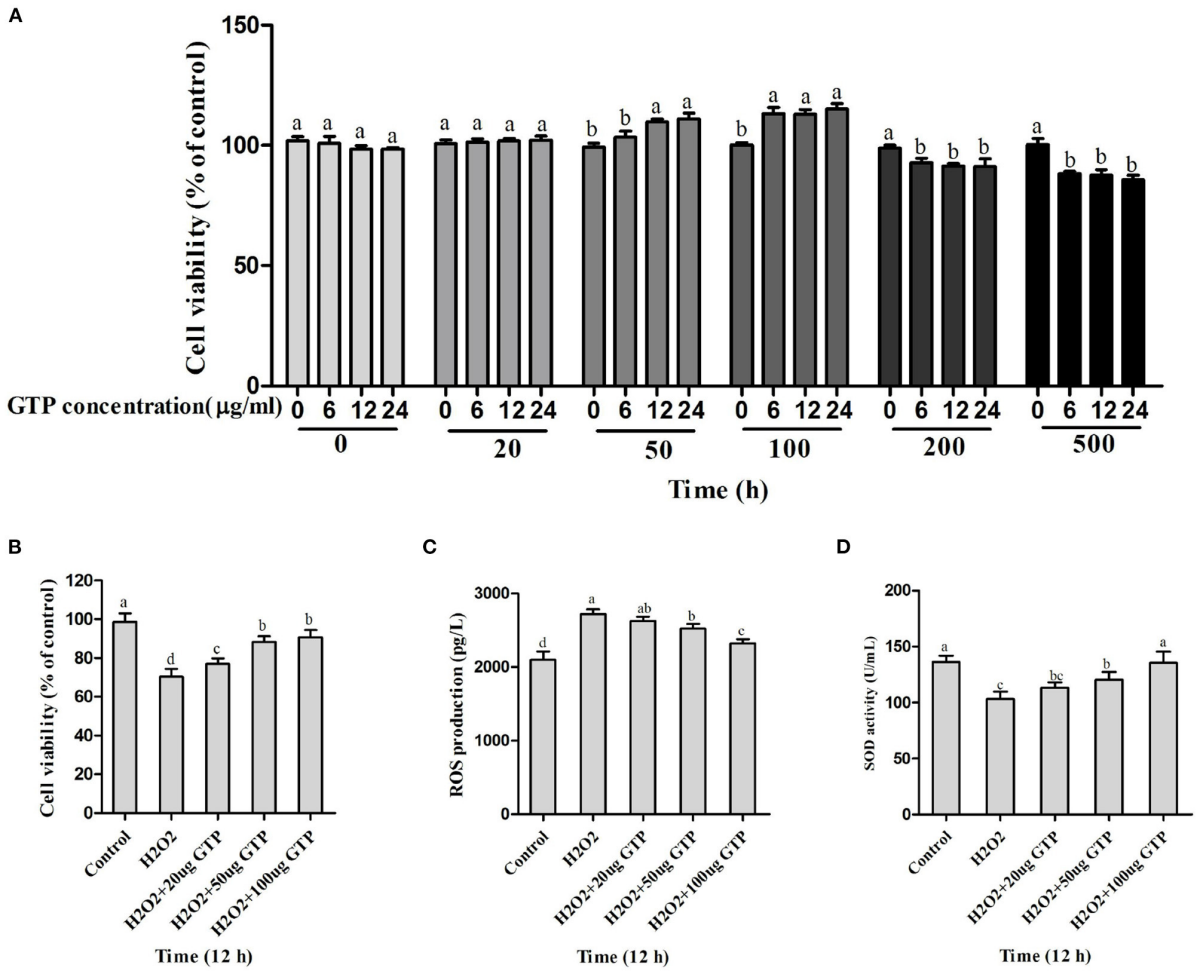
Screening for the safety concentration and time of GTP and optimum concentration of GTP to protect the BMECs. **(A,B)** Cell viability of BMECs, **(C)** ROS level in BMECs, and **(D)** SOD activity in BMECs. a,b,c,d, the adjacent lowercase letters indicate significant difference, the alternate lowercase letters indicate extremely significant difference, and the same lowercase letters indicate insignificant difference.

As shown in Figures 2A-D, with the increase in GTP concentrations (20–500 μg/ml) and incubation time (0–24 h), the viability of BMECs gradually increased. However, when the concentration of GTP exceeded 200 μg/ml, the viability of BMECs was significantly reduced (Figure 2A; *P* < 0.05). As shown in Figure 2, GTP alleviated the oxidative damage of BMECs caused by H_2_O_2_. With the increase in GTP concentration, cell viability (Figure 2B; *P* = 0.03) and SOD activity (Figure 2D; *P* = 0.02) increased significantly, while ROS concentration (Figure 2C; *P* < 0.01) decreased significantly. Therefore, 100 μg/ml of GTP and an incubation time of 12 h were selected for subsequent experiments.

### GTP Attenuated H_2_O_2_-Induced OS

**Figure 3:**
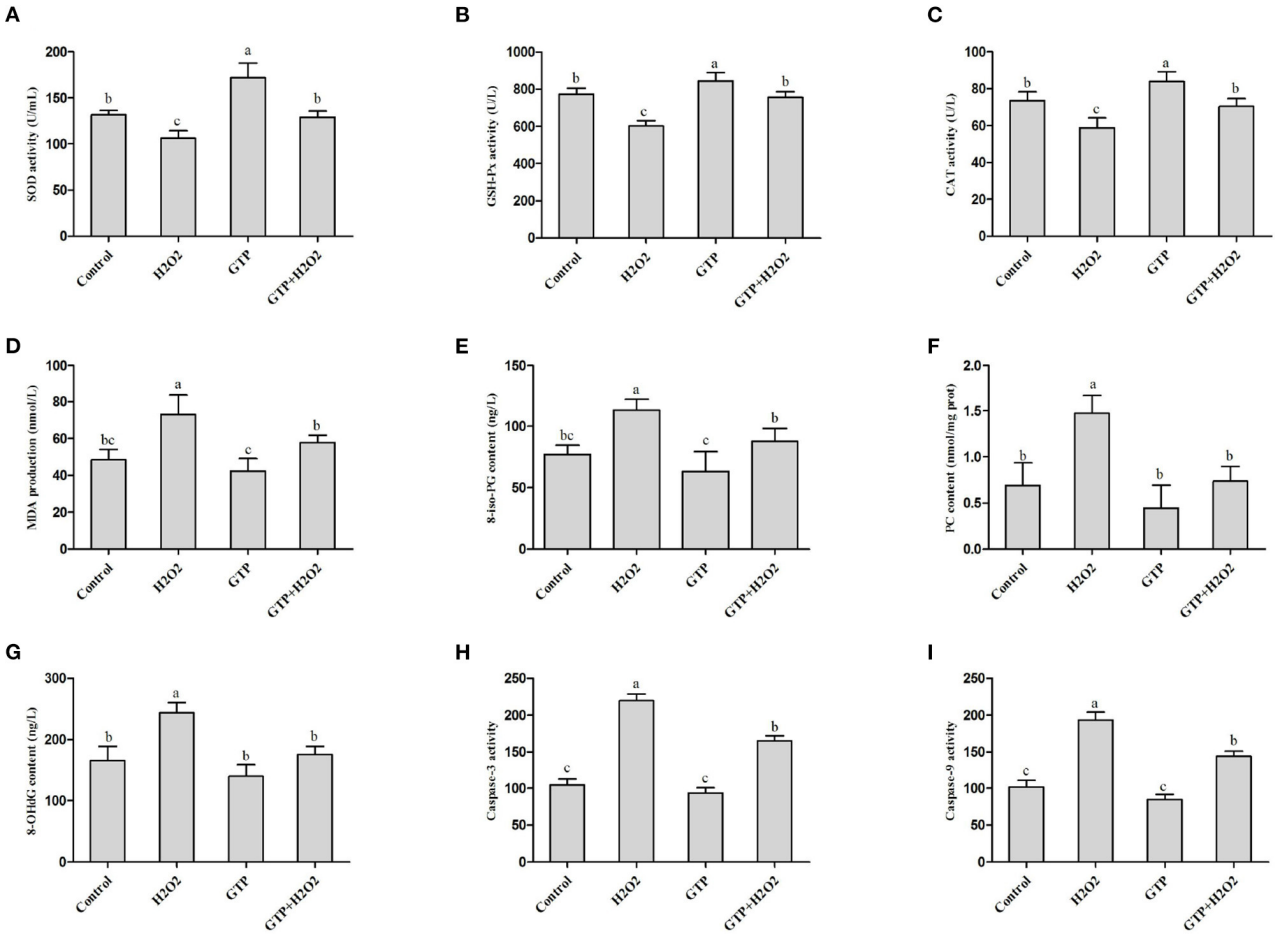
GTP attenuated H_2_O_2_-induced oxidative stress. **(A)** SOD activity, **(B)** GSH-Px activity, **(C)** CAT activity, **(D)** MDA content, **(E)** 8-iso-PG content, **(F)** PC content, **(G)** 8-OHdG contents, **(H)** caspase-3 activity, and **(I)** caspase-9 activity. a,b,c, the adjacent lowercase letters indicate significant difference, the alternate lowercase letters indicate extremely significant difference, and the same lowercase letters indicate insignificant difference.

Compared with the control group, the H_2_O_2_-treated group showed decreased activity of SOD (Figure 3A; *P* = 0.03), GSH-Px (Figure 3B; *P* = 0.02), and CAT (Figure 3C; *P* < 0.05) in BMECs but showed significantly increased content of MDA (Figure 3D; *P* < 0.05), 8-iso-PG (Figure 3E; *P* < 0.05), PC (Figure 3F; *P* < 0.05), and 8-OHdG (Figure 3G; *P* < 0.05) and increased activities of caspase-3 (Figure 3H; *P* = 0.01) and caspase-9 (Figure 3I; *P* = 0.01). However, the co-treatment of cells with GTP and H_2_O_2_ led to a significant increase in SOD, GSH-Px, and CAT activities (*P* < 0.05) and a significant decrease in MDA, 8-iso-PG, PC, and 8-OHdG contents and caspase-3 and caspase-9 activities compared to H_2_O_2_ treatment alone (*P* < 0.05).

### GTP Activated the NFE2L2 Signaling Pathway

**Figure 4:**
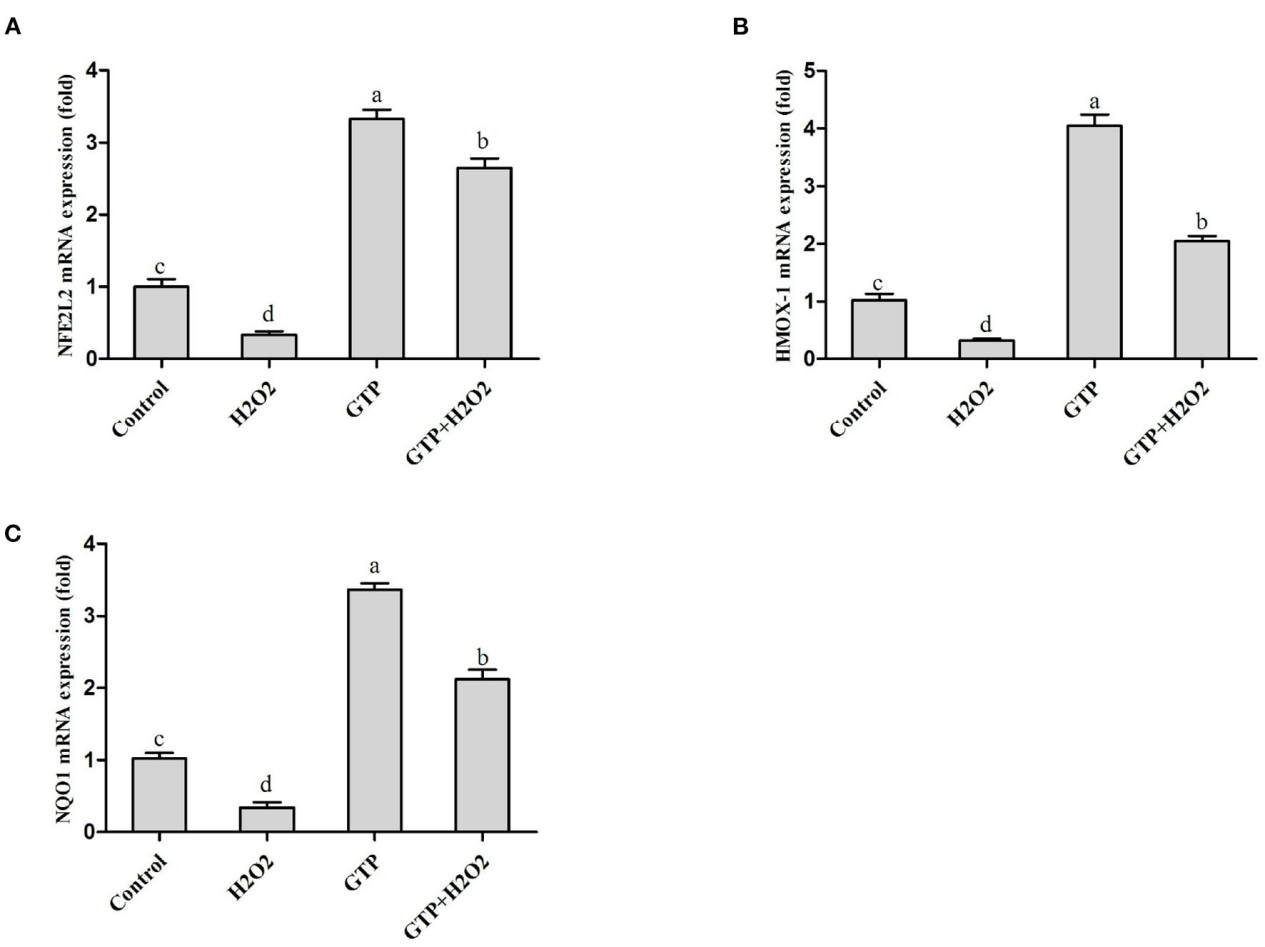
GTP activated the NFE2L2 pathway. BMECs were treated with H_2_O_2_ (500 μM) and/or GTP (100 μg/ml) for 12 h. **(A)** The mRNA expression level of NFE2L2 in BMECs, **(B)** the mRNA expression level of HMOX1 in BMECs, and **(C)** the mRNA expression level of NQO1 in BMECs. a,b,c,d, the adjacent lowercase letters indicate significant difference, the alternate lowercase letters indicate extremely significant difference, and the same lowercase letters indicate insignificant difference.

As shown in Figures 4A–C, compared with the control group, the H_2_O_2_-treated group showed significantly decreased mRNA expressions of NFE2L2 (Figure 4A; *P* = 0.03), HMOX1 (Figure 4B; *P* = 0.02), and NQO1 (Figure 4C; *P* = 0.04), while the GTP-treated group showed significantly increased mRNA expressions of NFE2L2 (*P* < 0.01), HMOX1 (*P* < 0.01), and NQO1 (*P* < 0.01). In addition, the co-treatment of cells with GTP and H_2_O_2_ led to a significant increase in the mRNA abundance of NFE2L2, HMOX1, and NQO1 compared to H_2_O_2_ treatment alone (*P* < 0.05).

### GTP Downregulated H_2_O_2_-Activated Caspase Pathway Activity

**Figure 5:**
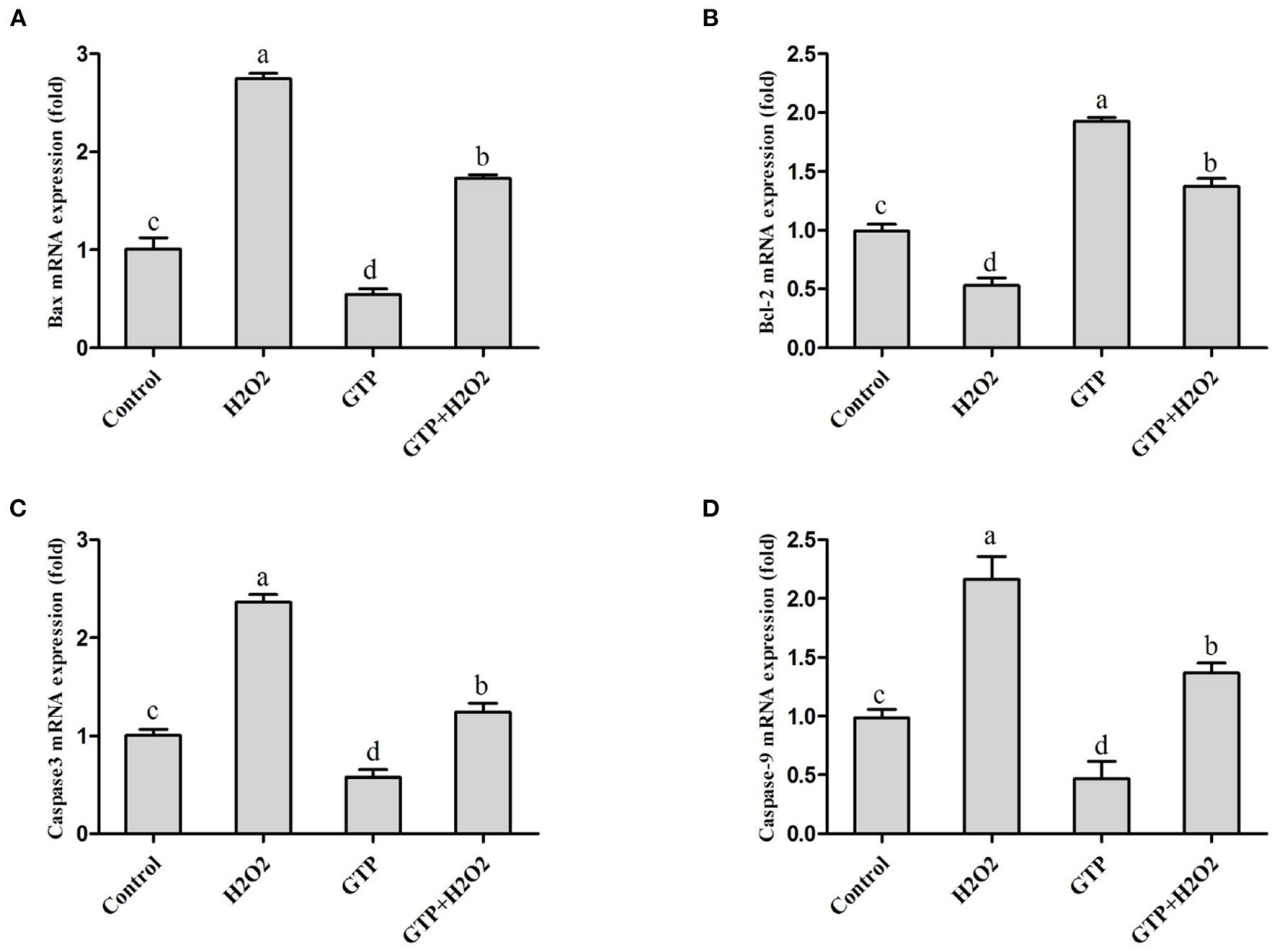
GTP attenuated the H_2_O_2_-activated caspase pathway and apoptosis. BMECs were treated with H_2_O_2_ (500 μM) and/or GTP (100 μg/ml) for 12 h. **(A)** Bax mRNA level in BMECs, **(B)** Bcl-2 mRNA level in BMECs, **(C)** Caspase-3 mRNA level in BMECs, and **(D)** caspase-9 mRNA level in BMECs. a,b,c,d, the adjacent lowercase letters indicate significant difference, the alternate lowercase letters indicate extremely significant difference, and the same lowercase letters indicate insignificant difference.

As shown in Figures 5A–D, culturing with H_2_O_2_ alone markedly increased the mRNA expression of Bax (Figure 5A; *P* < 0.01), caspase-3 (Figure 5C; *P* < 0.01), and caspase-9 (Figure 5D; *P* < 0.01), while it significantly decreased the mRNA expression of Bcl-2 (Figure 5B; *P* = 0.02). In contrast, GTP treatment significantly decreased the mRNA expressions of Bax (*P* = 0.04), caspase-3 (*P* = 0.03), and caspase-9 (*P* = 0.03) and significantly increased the mRNA expression of Bcl-2 (*P* < 0.01). Compared with the H_2_O_2_ treatment alone, the co-treatment of cells with GTP and H_2_O_2_ obviously increased the mRNA abundance of Bcl-2 (*P* < 0.05) and significantly decreased the mRNA abundance of Bax, caspase-3, and caspase-9 (*P* < 0.05).

### GTP Regulated the Expressions of TNF-α, IL-6, and IL-1β

**Figure 6:**
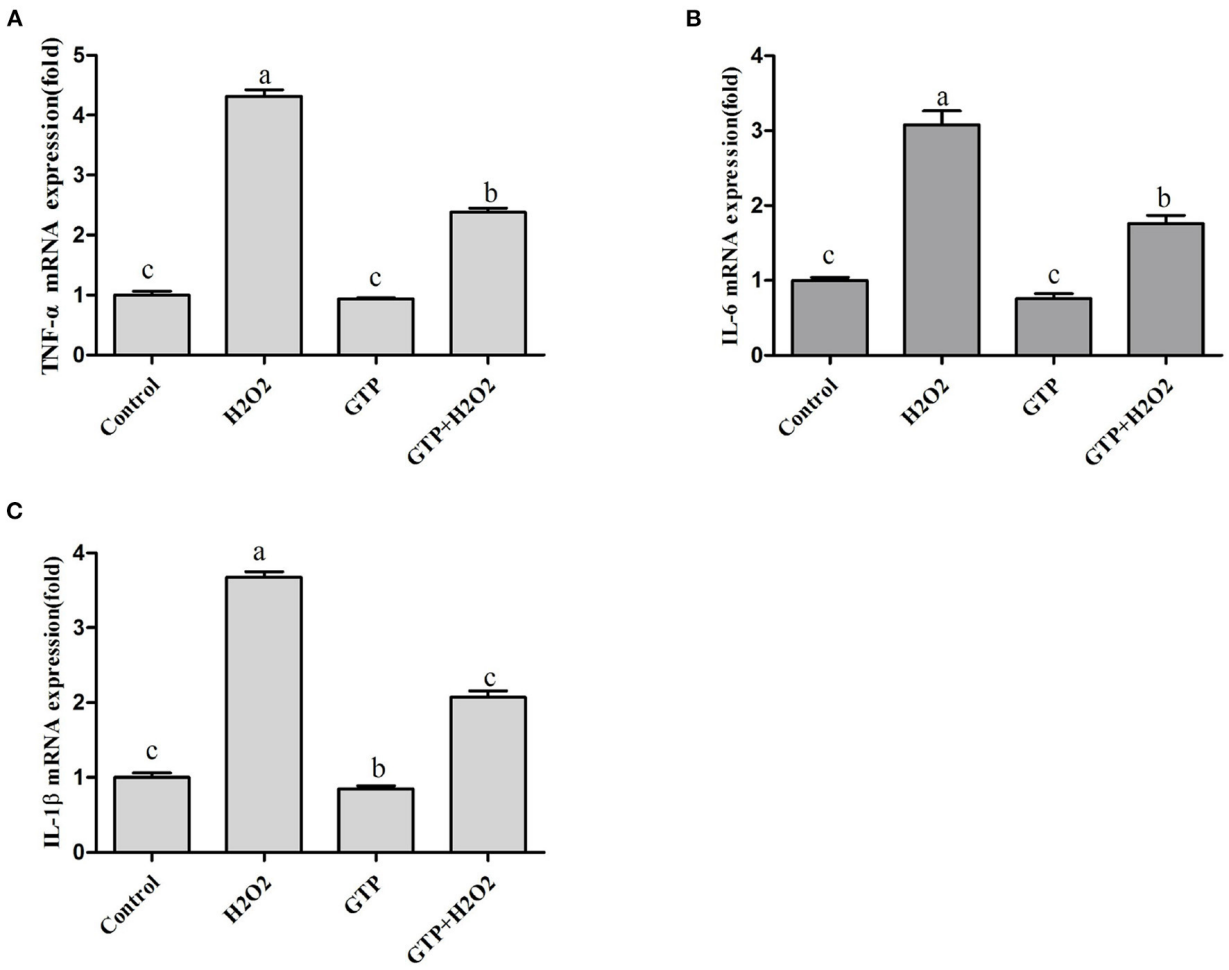
GTP regulated the expression of TNF-α, IL-6, and IL-1β. BMECs were treated with H_2_O_2_ (500 μM) and/or GTP (100 μg/ml) for 12 h. **(A)** TNF-α mRNA level, **(B)** IL-6 mRNA level, and **(C)** IL-1β mRNA level. a,b,c, the adjacent lowercase letters indicate significant difference, the alternate lowercase letters indicate extremely significant difference, and the same lowercase letters indicate insignificant difference.

As shown in Figures 6A–C, culturing with H_2_O_2_ alone markedly increased the mRNA expressions of TNF-α (Figure 6A; *P* = 0.02), IL-6 (Figure 6B; *P* = 0.04), and IL-1β (Figure 6C; *P* = 0.04), while GTP attenuated the H_2_O_2_-induced expression of inflammatory cytokines (*P* < 0.05).

### GTP Regulated the NFE2L2 Pathway Through the Mitogen-Activated Protein Kinase (MAPK) Signaling Pathway

**Figure 7:**
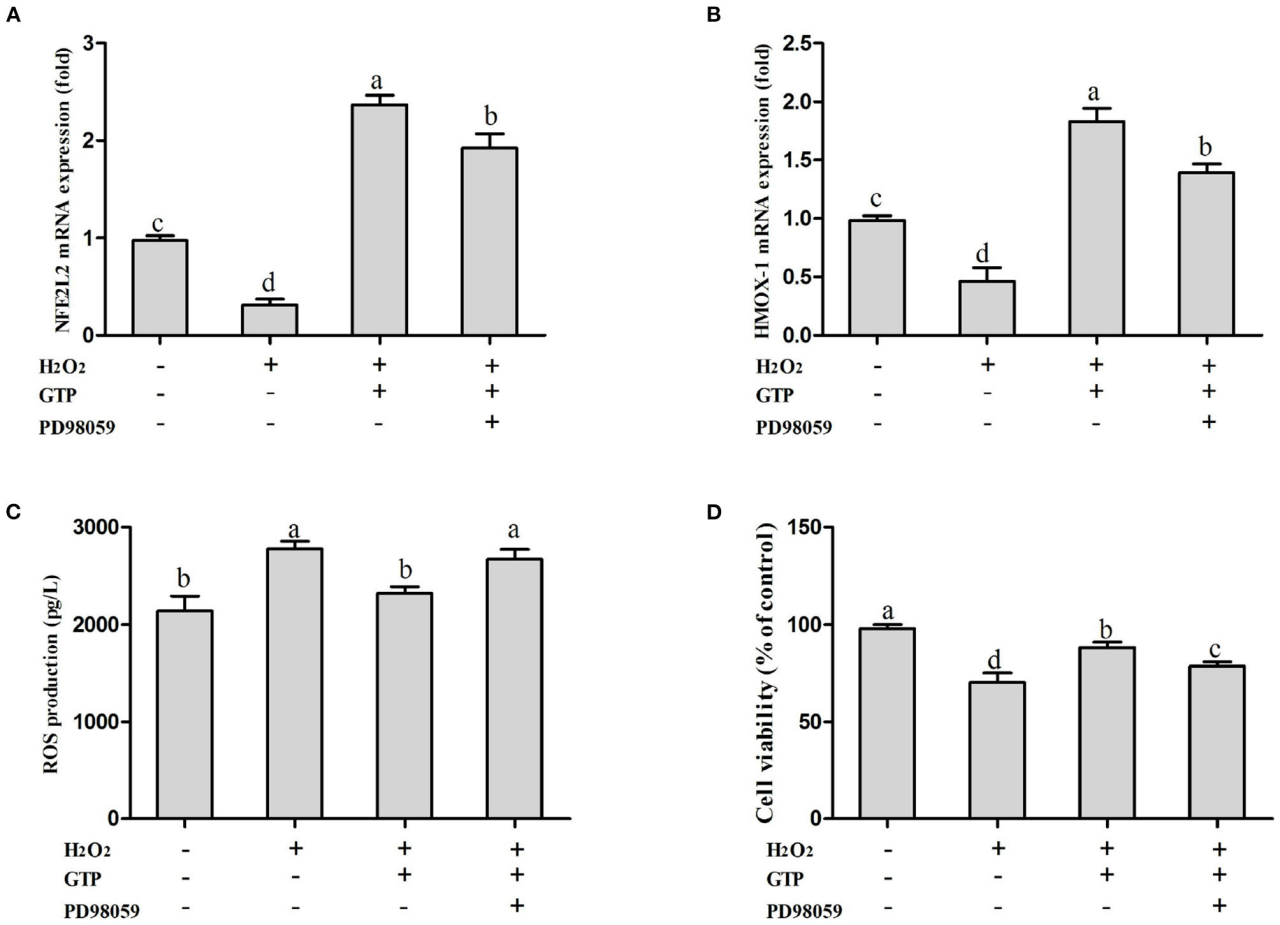
Inhibition on MAPK affected the activation effects of GTP on the NFE2L2 signaling pathway and the cell protection effects of GTP. **(A)** NFE2L2 mRNA level, **(B)** HMOX1 mRNA level, **(C)** ROS level, and **(D)** cell viability of BMECs. a,b,c,d, the adjacent lowercase letters indicate significant difference, the alternate lowercase letters indicate extremely significant difference, and the same lowercase letters indicate insignificant difference.

As shown in Figures 7A–D, compared with the H_2_O_2_-treated group, co-treatment with GTP + H_2_O_2_ significantly increased the mRNA expressions of NFE2L2 (Figure 7A; *P* < 0.01) and HMOX1 (Figure 7B; *P* < 0.01) and cell viability (Figure 7D; *P* < 0.01) and reduced ROS generation (Figure 7C; *P* = 0.03). However, GTP + H_2_O_2_ + PD98059 treatment greatly decreased the mRNA expressions of NFE2L2 and HMOX1 and cell viability (*P* < 0.05) and increased ROS (*P* < 0.05) generation compared with GTP + H_2_O_2_ treatment, suggesting that when H_2_O_2_ stimulated cells to cause OS, the phosphorylation of ERK1/2 played an important role in the rapid activation of the NFE2L2 signaling pathway by GTP.

### GTP Attenuated H_2_O_2_-Induced OS, Inflammatory Responses, and Apoptosis Through the NFE2L2 Signaling Pathway

**Figure 8:**
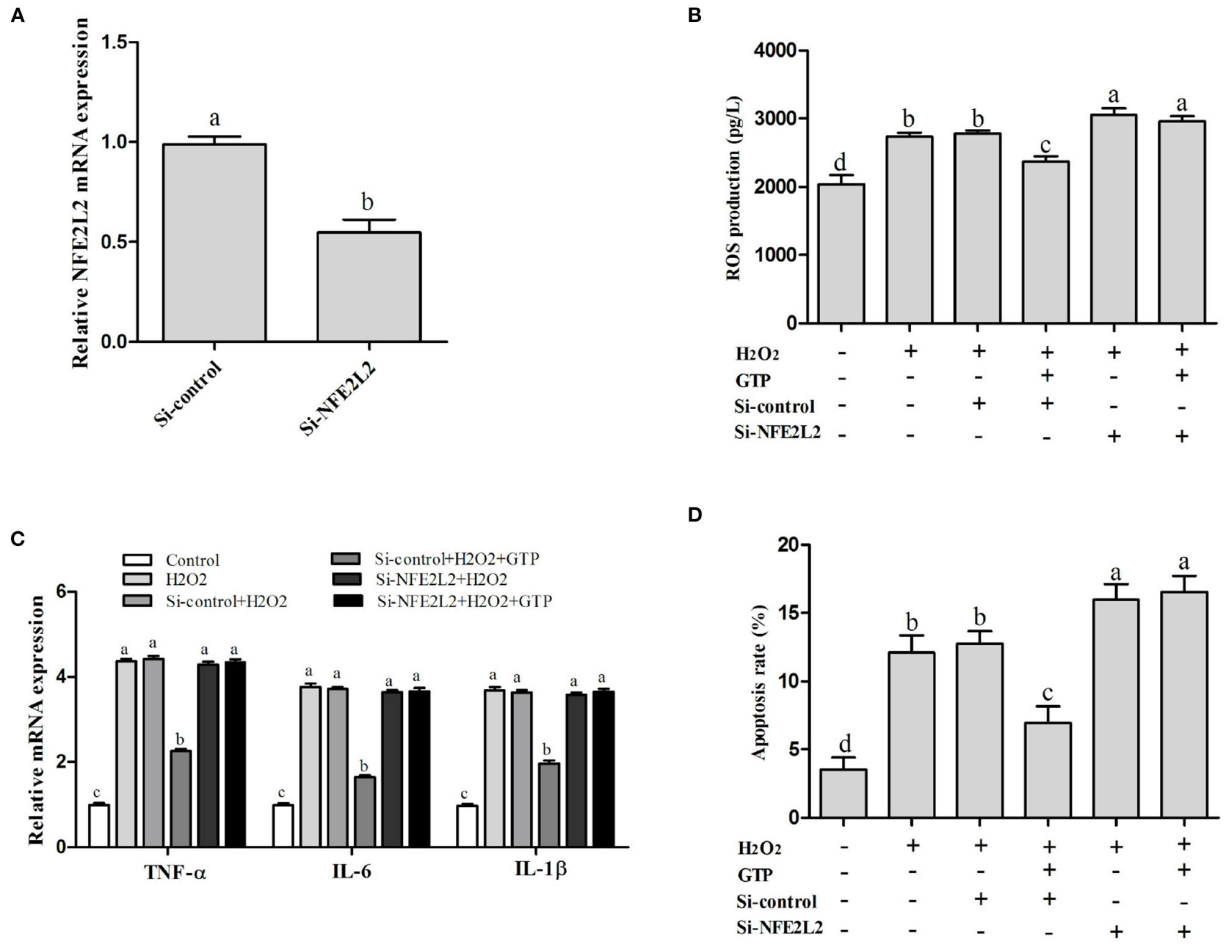
Knockdown of NFE2L2 attenuated the beneficial effects of GTP on H_2_O_2_-induced oxidative injury. **(A)** NFE2L2 mRNA level **(B)** ROS level; **(C)** TNF-α, IL-6, and IL-1β mRNA levels; and **(D)** the apoptosis rate of BMECs. a,b,c,d, the adjacent lowercase letters indicate significant difference, the alternate lowercase letters indicate extremely significant difference, and the same lowercase letters indicate insignificant difference.

Compared with that in the control group, the mRNA expression of NFE2L2 in the si-NFE2L2 group was significantly decreased (Figure 8A; *P* < 0.05). In addition, ROS generation in the group co-treated with GTP and H_2_O_2_ was significantly decreased compared to that in the H_2_O_2_-treated group (Figure 8B; *P* < 0.05). However, ROS generation in the si-NFE2L2 + GTP + H_2_O_2_-treated group was greatly increased compared with that in the GTP + H_2_O_2_-treated group (Figure 8B; *P* < 0.01). In addition, compared with the H_2_O_2_-treated group, the GTP + H_2_O_2_-treated group showed reduced mRNA expressions of TNF-α, IL-6, and IL-1β (*P* < 0.05). In contrast, compared to that in the GTP + H_2_O_2_-treated group, the mRNA expression of TNF-α, IL-6, and IL-1β was increased in the si-NFE2L2 + GTP + H_2_O_2_-treated group (Figure 8C; *P* < 0.05). Compared with the H_2_O_2_-treated group, the GTP + H_2_O_2_-treated group had a reduced apoptosis rate (Figure 8D; *P* < 0.05). In contrast, compared to that in the GTP + H_2_O_2_-treated group, the apoptosis rate was increased in the si-NFE2L2 + GTP + H_2_O_2_-treated group (Figure 8D; *P* < 0.01).

## Discussion

The mammary gland tissue is the most metabolic functional part in dairy cows. Due to the synthesis and secretion of large amounts of milk during lactation, aerobic metabolic activities are significantly increased, and there is a risk of excessive free radical generation. Excessive free radicals can cause oxidative damage, decrease the antioxidant function and immune function of dairy cow mammary epithelial cells, affect the normal lactation function of mammary cells, and finally lead to a decrease in milk production and milk quality (3, 14–16). Therefore, improving the antioxidant capacity of bovine mammary cells may be an effective therapeutic strategy against OS. GTP are natural flavonoids in tea. Studies have shown that the GTP has high antioxidant capacity *in vivo* and *in vitro* (10, 12, 17). In the present study, the data emphasized its biological potential against H_2_O_2_-induced OS and cell apoptosis. Its mechanism of action includes activating NFE2L2 and HMOX1 and NQO1 pathways and inhibiting the caspase/apoptosis pathways.

OS is caused by the imbalance between the formation of ROS and the reduction of antioxidant defense capabilities, which can cause substantial damage to nearby tissues, leading to the occurrence of diseases (18). Dairy cows in the transitional period will produce excessive ROS due to increased metabolic levels and increase the risk of cow mastitis and ketosis (17, 19, 20). Therefore, maintaining the intracellular redox balance and reducing the risk of diseases related to OS are extremely important for transitional dairy cows. Several recent studies suggested that GTP has high antioxidant activity and reduces the damage of OS. *In vivo* studies have shown that GTP can increase rat (21) and dairy cow (17) serum CAT, GSH-Px, and SOD levels and reduce the production of MDA. Liu et al. have also found that GTP can effectively reduce the oxidative damage caused by tributyltin (22). Our results suggested that H_2_O_2_ exceeding the physiological dose caused OS in BMECs, providing exogenous GTP significantly increased the SOD, GSH-Px, and CAT activities of BMECs, while reducing the content of ROS and MDA and the activities of caspase-3 and caspase-9. In addition, under OS conditions, the decrease in PC, 8-OHdG, and 8-iso-PG concentrations also confirmed that the positive effect of GTP on OS extended to the reduction in protein, DNA, and lipid damage.

The NFE2L2 signaling pathway plays a key role in maintaining cell redox balance. Schogor et al. found that the abundance of NFE2L2 mRNA in the mammary glands of cows supplemented with flax meal increased linearly, indicating that NFE2L2 may be involved in promoting the antioxidant capacity of BMECs (23). Previous studies have shown that increasing the NFE2L2 mRNA expression can increase the cell survival rate under OS conditions, and the activation of the NFE2L2 pathway is the key to the upregulation of antioxidant enzyme expression (24). In addition, in several cell models, GTP can activate the NFE2L2 pathway by disrupting NFE2L2/keap1 (25). Our results found that GTP can activate the NFE2L2 pathway in H_2_O_2_-damaged BMECs and increase the expression of HMOX1 and NQO1, which is consistent with the study by Ma et al. (11) and Song et al. (26). This indicated that GTP can alleviate OS by increasing the expression of NFE2L2, HMOX1, and NQO1 in BMECs and protect BMECs from H_2_O_2_-induced OS.

Some studies show that OS can induce cell apoptosis (27, 28). Apoptosis is the active death process of cells under the activation, expression, and regulation of a series of genes. There are mainly two classic apoptosis signal transduction pathways, namely, the cell surface death receptor pathway and the mitochondrial pathway. The caspase pathway plays a key role in controlling the apoptotic pathway inherent in the mitochondrial initiator caspase component of the apoptosome complex, and caspase-9 can be activated by ROS and therefore plays a vital role in activating the effector caspase in response to various death stimuli. Activated caspase-9 can activate caspase-3 zymogen to form caspase-3 with protein-decomposing activity, thereby initiating the caspase cascade reaction and leading to cell apoptosis (29–31). In addition, the mitochondrial pathway also includes Bcl-2 family proteins. Bcl-2 can prevent cell apoptosis by inhibiting membrane damage mediated by oxygen free radicals and stabilizing the mitochondrial membrane potential (32). The Bax gene belongs to the Bcl-2 gene family. The encoded Bax protein can form a heterodimer with Bcl-2 and inhibit Bcl-2, thereby promoting cell apoptosis (33). Therefore, in this study, by detecting the gene expression of caspase-3, caspase-9, Bax, and Bcl-2, it is possible to reveal the molecular mechanism by which tea polyphenols alleviate cell apoptosis induced by OS. Present results indicated that OS activated the mitochondrial apoptotic pathway, leading to the activation of caspase-3 and cell apoptosis. GTP can inhibit the activation of caspase-3 and the expression of Bax and increase the mRNA expression of Bcl-2, thereby inhibiting the occurrence of BMECs apoptosis caused by OS.

In addition to inducing cell apoptosis, OS also causes inflammation. During the perinatal period of dairy cows, OS is closely related to inflammation (34). Moreover, OS and systemic inflammation may be the main factors leading to metabolic disorders and diseases in perinatal dairy cows. Studies have shown that ROS can cause excessive activation of the NF-κB signaling pathway, which may increase the expression of inflammation-related cytokines and cause the inflammatory response (35). Studies have shown that OS can increase the expression of inflammatory cytokines such as TNF-α, IL-6, and IL-1β in dairy cow mammary epithelial cells (28). Our results showed that H_2_O_2_-induced OS in BMECs increases the mRNA expressions of TNF-α, IL-6, and IL-1β, and GTP could inhibit TNF-α, IL-6, and IL-1β mRNA expressions. This indicated that GTP can reduce ROS-induced inflammation in BMECs by inhibiting the expression of inflammatory cytokines.

Previous data showed that activation of the NFE2L2-antioxidant signaling pathway attenuated OS and apoptosis (11, 36–38). In this study, when NFE2L2 was silenced, the antioxidant function of GTP was strongly inhibited (i.e., the level of ROS in BMECs was significantly increased). In addition, after the NFE2L2 gene was silenced under OS, GTP failed to reduce the apoptotic rate of BMECs, which further indicated that the NFE2L2 pathway is very important for GTP to protect BMECs against OS induced by H_2_O_2_. As reviewed, our results confirmed the direct role of GTP and NFE2L2 in the antioxidant response.

In addition, many cellular kinases, including MAPK, may regulate NFE2L2. MAPK is involved in the upregulation of antioxidant/detoxification enzyme activity (39) and, together with the induction of NFE2L2, constitutes an important way to protect cells from OS (40). The MAPK pathway-related signals is a family of highly conserved protein kinases (41). The activation of this system has an important regulatory effect on cell growth, differentiation, and response to stress. Studies have shown that antioxidants can activate the NFE2L2 pathway and increase the mRNA expression of HMOX1 by activating the ERK1/2 and PI3K pathways (42). In this study, the results show that GTP can increase the mRNA expression of the NFE2L2 and HMOX1 in H_2_O_2_-induced oxidative damage in BMECs, reduce ROS production, and increase cell viability. When PD98059 was used to inhibit the mRNA expression of ERK1/2, it downregulated the expression of the NFE2L2 pathway and its downstream gene HMOX1 and increased ROS production and reduced cell viability. Therefore, the current data in this study indicated that GTP may protect BMECs from OS by activating the ERK1/2 pathway.

## Conclusion

In summary, the results of this study provide important evidence for the potential cytoprotective effect of GTP against H_2_O_2_-induced oxidative damage in BMECs. The mechanism of action included reducing ROS production and the gene expression of cytokines, maintaining intracellular redox balance and inflammatory balance, and activating the NFE2L2 pathway and ERK1/2 pathway, while inhibiting the caspase/Bax apoptosis pathway. The cytoprotective effect of GTP depended at least in part on the activation of the ERK1/2–NFE2L2–HMOX1 signaling pathway. Furthermore, our data indicated that GTP may be used as an antioxidant drug and inflammation inhibitor for dairy cows during the transition period.

## Data Availability Statement

The original contributions presented in the study are included in the article/supplementary material, further inquiries can be directed to the corresponding author/s.

## Author Contributions

YM wrote the first version of the manuscript with assistance from XM, YA, YS, and WD. ML assisted in the writing and comments of the manuscript. HB and CZ participated in the cell culture experiment. All authors contributed to the article and approved the submitted version.

## Funding

This work was supported by grants from the National Natural Science Foundation of China (No. 32060765, Beijing, China), Ningxia University Scientific Research Start-up Project (No. 030900002154), Ningxia Natural Science Foundation of China, Science and Technology Planning project of Inner Mongolia Autonomous Region (No. 2021GG0025), Inner Mongolia Natural Science Foundation of China (No. 2019BS03033, Hohhot, China), and Inner Mongolia Academy of Agricultural & Animal Husbandry Science Innovation Foundation (No. 2019CXJJM06, Hohhot, China).

## Conflict of Interest

The authors declare that the research was conducted in the absence of any commercial or financial relationships that could be construed as a potential conflict of interest.

## Publisher's Note

All claims expressed in this article are solely those of the authors and do not necessarily represent those of their affiliated organizations, or those of the publisher, the editors and the reviewers. Any product that may be evaluated in this article, or claim that may be made by its manufacturer, is not guaranteed or endorsed by the publisher.
